# Impact of COVID-19 on BISE Research and Education

**DOI:** 10.1007/s12599-020-00666-9

**Published:** 2020-09-01

**Authors:** Wil van der Aalst, Oliver Hinz, Christof Weinhardt

**Affiliations:** 1 Lehrstuhl für Informatik 9, RWTH Aachen, Ahornstr. 55, 52056 Aachen, Germany; 2 Faculty of Economics and Business Administration, Goethe University Frankfurt, Theodor-W.-Adorno-Platz 4, 60323 Frankfurt am Main, Germany; 3 Institute of Information and Systems and Marketing (IISM), Karlsruhe Institute of Technology (KIT), Kaiserstr. 89, 76131 Karlsruhe, Germany

## Introduction

The last BISE Editorial board meeting took place on 8 March 2020 in Potsdam and was co-located with the International Conference on Wirtschaftsinformatik (WI2020). This was just 1 week before COVID-19 restrictions were imposed in Germany. It was, by the way, also our first Editorial Board Meeting held in a hybrid (analog and digital) mode. One day later, on March 9, the first coronavirus death in Germany was reported, and on the same day, the government of Italy imposed a national quarantine. WI 2020 was probably one of the last major conferences to take place in 2020 in the usual (i.e., face-to-face) form. In the weeks following our BISE Editorial board meeting, universities and schools were closed and conferences were canceled or converted into online events. The way the COVID-19 pandemic was handled varied from county to country, but it dramatically impacted every individual and every organization on the globe. Next to the devastating short-term effects, we believe that there will also be longer-term effects on education and scientific gatherings. It will permanently change the way we work, educate, interact, and do business (the “new normal”). Therefore, in this editorial, we reflect on the effects of COVID-19 on BISE Research and Education.

## Impact of COVID-19 on Education

In most countries, primary and secondary schools closed temporarily. Universities had to switch to online education. This impacted children, students, parents, and teachers, and exposed the shortage of computers among the different groups. According to UNESCO, the COVID-19 pandemic has interrupted classroom learning for at least 9 out of 10 students worldwide, and about half of the students worldwide have no access to online teaching (826 million learners worldwide have no access to a computer) (UNESCO 2020). The “digital divide” between rich and poor is disturbing. For higher education, the transition was more straightforward. Many universities were able to switch to online education in just a few weeks. However, also in higher education experiences are mixed.

On the one hand, students could study in a more flexible, self-paced manner. On the other hand, social interaction is a crucial ingredient for studying. Universities and college campuses are unique ecosystems where students with different backgrounds learn and socialize in close proximity to each other. In some countries, the percentage of international students is well over 25%. Hence, there will also be a longer-lasting economic effect on universities in some countries.

It is to be expected that hybrid forms of teaching will become the new norm. Novel ways of combining face-to-face and online teaching need to be explored. The challenge is to become more robust when a new lockdown occurs. A significant hurdle for online education is the testing of students. Online proctoring is far from trivial. How to ensure that the right person is doing the test and does not have access to teaching material or other sources of information? Proctoring may raise privacy concerns. For example, student councils in the Netherlands tried to stop the use of Proctorio and other online proctoring tools at Dutch universities. Privacy and safety concerns are also blocking interaction in virtual classrooms.

Unsurprising, the usage of Zoom, Skype, Cisco Webex, Slack, and Microsoft Teams increased dramatically. In March 2020, the number of downloads of the video conferencing system Zoom increased by 728% (Sloan 2020). The number of daily active users of Microsoft Teams grew from 32 million in March to 75 million in April. This reflects the major changes in communication in educational settings and beyond.

## Impact of COVID-19 on Research and Scientific Conferences

Many researchers are also involved in teaching, and the transition to online formats required additional efforts. As a result, less time could be spent on research. Although for some academics home-office helped to focus, for most researchers, group processes and feedback are crucial. Bachelor, Master, and PhD student supervision generally suffered from the situation. In Knoben and Oerlemans (2006), the authors review literature on the effects of proximity on inter-organizational collaboration. Many studies show that, the larger the distance between workers, the more difficult it is to transfer knowledge and experience. Of course, this also applies to research collaboration. New ideas typically emerge through interactions, and thinking errors often only reveal themselves after presentations and discussions. For some scientific projects, it also became harder to collect data, especially in experimental settings and field studies that involve human interaction. As participants could not be invited to labs and other facilities, several projects had to been paused for a yet undefined period. Collecting data in observed environments or with physio-sensors became significantly harder or almost impossible. Interviews had to be moved to online formats. Right now, it is not clear how these restrictions will affect future scientific insights (and maybe even scientific careers). If the current situation remains like this or will occur more often in the future, new ways of conducting experimental research have to be evaluated. In our last editorial (Weinhardt et al. 2020), we discussed the application of citizen science in Information Systems research. As many aspects of life (work, schooling, social interaction) moved back into the homes of citizens, this now seems to be more current and relevant than ever before.

Most conferences have switched to online formats. Examples of scientific conferences in the field relevant for the BISE community that made the switch include CAiSE, BPM, CBI, ICPM, ECIS, DATA, ICSOFT, PACIS, AMCIS, BIS, ICDE, and ICIS. Very few were canceled or postponed. In most cases, (short) presentations were provided online. The online format, in principle, allowed more people to attend, but in most cases, truly active participation was reduced significantly. Most participants would cherry-pick selected talks and multitask while watching. Some conferences also experimented with new formats such as 180-s video teasers of each paper. The conference proceedings were typically published as usual.

Also, business-oriented conferences had to resort to online formats. For example, in the process mining space, both the annual Process Mining Camp (organized by Fluxicon) and Celosphere (organized by Celonis) moved to online formats. In 2019, Celosphere attracted about 1000 participants (face-to-face). Celosphere Live had 18,000 registrants and 8600 actual attendees (April 2020). Process Mining Live (organized by IQPC/PEX) and several other online process mining conferences had hundreds of participants. This shows that the potential reach of online events may be much higher than face-to-face events.

Many organizations realized that it is relatively easy to organize webinars and other online events (see for example, ABBY Expert talks and PAFnow AprilTalks). Despite the various successful formats, interaction is very different. Virtual conferences seem a poor substitute for face-to-face conferences due to the lack of social interactions during coffee breaks, offline discussions, and breakouts sessions. Virtual coffee breaks, “snackable sessions”, and other ideas try to overcome the inherent limitations, but with limited success until now. Nevertheless, experiences with online conferences might cause organizations to question the need to send employees off on international travel, given the focus on sustainability and cutting carbon emissions. Like in teaching, conferences may shift to more hybrid formats.

## Impact of COVID-19 on Businesses

At this time, the impact on the global economy is unclear and may change quickly (e.g., due to the potential availability of new vaccines). However, the pandemic will accelerate some of the pre-existing trends and create new ones. In addition, business models will have to evolve in order to be COVID-proof. Online sales increased while “brick-and-mortar” sales decreased. According to Bradley et al. (2020), the gap in economic profit between the top-performing companies and other companies else has widened dramatically. Between December 2018 and May 2020, the top quintile of companies grew its total market-implied annual economic profit by $335 billion, while companies in the bottom quintile lost a $303 billion (Bradley et al. 2020).

The crisis also showed the vulnerability of supply chains. When borders close, it is impossible to replenish specific products, and it is very difficult to manage the increased demand for medicine, masks, toilet paper, etc. Hence, some countries will reconsider where to produce certain items. Larger Original Equipment Manufacturers (OEMs, e.g., in the automotive industry) rely on hundreds of Tier 1 suppliers and indirectly on thousands of Tier 2 suppliers. Due to the problems experienced, “supply chain resilience” will be an important topic in the coming years.

Inside organizations, there will be a redistribution of work and streamlined decision-making processes. Some organizations were able to operate in a reasonable manner with just a fraction of the normal personnel working. In some organizations, changes could be implemented that would normally take years. Other organizations went bankrupt due to a lack of agility. In addition, the limits of working remotely have become clear. This is illustrated by the fact that people remaining in the office tend to do the non-routine work normally done by others. Due to all these experiences, office and work life will not be the same after COVID-19 (Boland et al. 2020).

## Scientific Challenges Put Forward by the COVID-19 Pandemic

COVID-19 has accelerated and will accelerate digitalization initiatives (Bradley et al. 2020). The importance of operational processes and supporting information systems has been demonstrated by the crisis. The lack of reliable statistics and the inability to respond quickly enough at the beginning of the pandemic provided important lessons. The lack of reliable data and the inability to understand simple mathematical models (e.g., the Kermack–McKendrick and Reed–Frost epidemic models) lead to delayed political decisions. However, the problems experienced also pose interesting new scientific challenges.

Research in each of the BISE departments can contribute to better handling global shocks such as the current pandemic.

- The *Business Process Management* Department (Jörg Becker and Jan Mendling). Business Process Management (BPM) initiatives drive intentional change, but should also cope with unintentional change. Research should show how process models and process-aware information systems can help to improve business processes in times of radical changes. BPM can also help to better organize health-care processes and use the available resources in the best way possible.
- The *Decision Analytics and Data Science* Department (Natalia Kliewer and Stefan Lessmann**).** The importance of quantitative methods, business analytics, computational logistics, and network management was illustrated by the crisis. Having the right data and making the right decisions under time pressure requires a proper data-science pipeline. Moreover, decision support needs to deal with “wicked problems” that may have no “acceptable” solution.
- The *Digital Business Management and Digital Leadership* Department (Jens Dibbern and Roman Beck). The pandemic will fuel a new digitization wave. Research in this department may help to successfully realize the required digital transformation. Moreover, the pandemic may help to remove obstacles to blockchain adoption. Blockchain technologies are suited to verifying, securing, and sharing data, and thus, ideal for managing multi-party, inter-organizational, and cross-border transactions in times of COVID-19.
- The *Economics of Information Systems* Department (Dennis Kundisch and Kai Lung Hui). Many organizations will need to reconsider their business models. Electronic markets and digital platforms in times of Corona need to be supported by new economic theories and methods. An evaluation of the mitigation policies used by different governments may help to respond better. Economic models may also be adapted to better capture risks and resilience.
- The *Enterprise Modeling and Business Ecosystems Department* (Dimitris Karagiannis and Jelena Zdravkovic). Model-based development and the evolution and evaluation of enterprise-wide or business ecosystem-wide information systems are important in times where the context of these systems is rapidly changing. Domain-specific models, conceptual/reference modeling, and enterprise architecture management can help to structure future-proof information systems.
- The *Human Computer Interaction and Social Computing* Department (Alexander Mädche, Stefan Stieglitz, and Shirley Gregor). The department deals with research focusing on the design and use of interactive technologies that affect individuals, groups, organizations, communities, and networks. For sure, COVID-19 is changing the way that people interact with other people (e.g., co-workers) and information systems.
- The *Information Systems Engineering and Technology* Department (Óscar Pastor) The department deals with data and software engineering methods. Data management (e.g., supporting data lakes) and the ability to quickly develop software systems are vital in times of radical change.

The above list shows that BISE research can help to better deal with the effects of COVID-19. Moreover, the pandemic also provides new scientific challenges. This offers a unique opportunity to show the relevance of BISE in education and research (face-to-face or online). Until July 2020, 4 manuscripts relating to pandemics have been submitted to BISE by the community, treated by our editorial board with special treatment regarding process times. Two are likely to become published, thus illustrating the eagerness and ability of our community to address contemporary challenges.
